# Advanced Optical Information Encryption Enabled by Polychromatic and Stimuli‐Responsive Luminescence of Sb‐Doped Double Perovskites

**DOI:** 10.1002/advs.202308390

**Published:** 2024-04-16

**Authors:** Yijun Zhao, Xingru Yang, Lun Yang, Fangjian Xing, Cihui Liu, Yunsong Di, Guiyuan Cao, Shibiao Wei, Xifeng Yang, Xiaowei Zhang, Yushen Liu, Zhixing Gan

**Affiliations:** ^1^ Center for Future Optoelectronic Functional Materials School of Computer and Electronic Information/School of Artificial Intelligence Nanjing Normal University Nanjing 210023 China; ^2^ Institute for Advanced Materials Hubei Key Laboratory of Pollutant Analysis & Reuse Technology Hubei Normal University Huangshi 435002 China; ^3^ Nanophotonics Research Center Shenzhen Key Laboratory of Micro‐Scale Optical Information Technology Shenzhen University Shenzhen 518060 China; ^4^ College of Electronic and Information Engineering Changshu Institute of Technology Suzhou 215500 China; ^5^ Department of Electrical Engineering and Computer Science Ningbo University Ningbo 315211 China

**Keywords:** double perovskite, information encryption, multi‐color luminescence, stimuli‐responsive luminescence

## Abstract

The smart materials with multi‐color and stimuli‐responsive luminescence are very promising for next generation of optical information encryption and anti‐counterfeiting, but these materials are still scarce. Herein, a multi‐level information encryption strategy is developed based on the polychromatic emission of Sb‐doped double perovskite powders (SDPPs). Cs_2_NaInCl_6_:Sb, Cs_2_KInCl_6_:Sb, and Cs_2_AgInCl_6_:Sb synthesized through coprecipitation methods exhibit broadband emissions with bright blue, cyan, and orange colors, respectively. The information transmitted by specific SDPP is encrypted when different SDPPs are mixed. The confidential information can be decrypted by selecting the corresponding narrowband filter. Then, an encrypted quick response (QR) code with improved security is demonstrated based on this multi‐channel selection strategy. Moreover, the three types of SDPPs exhibit three different water‐triggered luminescence switching behaviors. The confidential information represented by Cs_2_NaInCl_6_:Sb can be erased/recovered through a simple water spray/drying. Whereas, the information collected from the green channel is permanently erased by moisture, which fundamentally avoids information leakage. Therefore, different encryption schemes can be designed to meet a variety of encryption requirements. The multicolor and stimuli‐responsive luminescence greatly enrich the flexibility of optical information encryption, which leaps the level of security and confidentiality.

## Graphical abstract

An information encryption strategy is developed based on the polychromatic and hydrochromic luminescence of Sb‐doped double perovskite powders (SDPPs). The confidential information can be decrypted by selecting the correct filter. Moreover, the SDPPs exhibit hydrochromic behaviors, implying the confidential information can be erased/recovered by tuning moisture. Therefore, different encryption schemes can be design for various requirements.

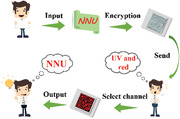

## 1 Introduction

Now, the huge amounts of information have reformed the daily life of human beings. However, a large number of false information and fake commodities that are difficult to discern from the truth are posing a potential threat to human security and social stability.^[^ ^1^, ^2^, ^3^, ^4^, ^5^ ^]^ Therefore, it is urgent to develop advanced information encryption and anti‐counterfeiting technology. Optical anti‐counterfeiting, especially fluorescent anti‐counterfeiting has the characteristics of high visibility, simple design, low‐cost fabrication, and convenient validation.^[^ ^6^, ^7^, ^8^, ^9^, ^10^ ^]^ However, the current fluorescent anti‐counterfeiting labels are prone to theft, imitation, or forgery owing to the increasing familiarity of counterfeiters with existing fluorescence ones.

The multiplexing of luminescence parameters, including both spectral and temporal dimensions, is a promising approach to increase optical information storage capability and encryption security.^[^ ^11^, ^12^, ^13^, ^14^, ^15^, ^16^ ^]^ By using different lanthanide ions, Li et al precisely tailored the emission colors of hydrogels in a wide spectrum range. The hydrogel blocks with various luminescence emission colors (i.e., red, green, yellow, and colorless) were assembled into a 3D array for confidential information protection.^[^ ^17^ ^]^ In our previous report, we co‐multiplexed spectral and temporal dimensions for optical encoding based on photoluminescence (PL) and persistent‐luminescence (PersL) at four different wavelengths. The information is unable to be decoded until both PL and PersL spectra are collected, suggesting a substantial improvement in information security and the security level of anti‐counterfeiting.^[^ ^18^ ^]^ On the other hand, external stimuli‐responsive luminous states can store and deliver information in a swappable manner. Thus, the confidential information can be hidden. Liang et al. demonstrated a time division duplexing based on eco‐friendly carbon nanodots (CNDs).^[^ ^19^ ^]^ Luminescence lifetimes of the bare CNDs can be manipulated from nanosecond to second scale by introducing water, while the PL lifetime of the CNDs confined in a silica shell keeps unchanged. Thus, spatial‐temporal overlapping information based on the CNDs and CNDs@silica can be resolved by time division duplexing.^[^ ^19^ ^]^ These novel designs based on multi‐color or stimuli‐responsive luminescence show great potential as multilevel anti‐counterfeiting solution. Logically, co‐multiplexing polychromatic and stimuli‐responsive characters can further improve the security, which is urgently desirable for new generation of anti‐counterfeiting technology. However, smart materials with these two characters remains to be explored.

Recently, lead‐free halide double perovskites (LHDPs) draw increasing attention as emerging luminescent materials due to their inherent high absorption coefficients, low trap densities, low‐toxicity, good stability, and high PL quantum yield (PLQY).^[^ ^20^, ^21^, ^22^, ^23^, ^24^ ^]^ On the one hand, LHDPs can form different electronic dimensions by changing the connectivity of halide octahedra. On the other hand, different ions, such as rare earth ions and ions with ns^2^ outer electronic configuration, can be compatibly doped into the LHDP crystal structure.^[^ ^25^, ^26^, ^27^, ^28^, ^29^ ^]^ The various electronic dimensions and versatile doped ions provide abundant opportunities for creating multistate and switchable luminescent behaviors. For instance, Zhu et al reported a rare earth doped LHDP Cs_2_NaEr_0.3_Yb_0.7_Cl_6_, which exhibited bright red and yellow under UV and NIR laser excitation, respectively. Then, the high‐level anti‐counterfeiting and information encryption applications are proposed based on dual‐mode polychromatic emission.^[^ ^30^ ^]^ Hydrochromic behavior that change the luminescence color upon exposure to moisture also has been demonstrated in LHDPs due to the transition from 3D to 0D electronic dimensions. Han et al reported Cs_3_GdCl_6_ metal halide as a host for photon up‐conversion. The Cs_3_Gd_(1−x−y)_Yb_x_Er_y_Cl_6_ photonic crystals (PCs) exhibited moisture‐sensitive hydrochromic up‐conversion luminescence (UCL) properties. Upon exposure to atmospheric moisture, the UCL of CGC:Yb^3+^, Er^3+^ PCs changes from green to red. Then, this hydrochromic UCL color change was further exploited for information encryption by laser handwriting.^[^ ^31^ ^]^ However, until now, information encryption based on LHDPs was yet limited to two emissive states or two emission colors, restricting the security level.

In this work, a high‐level encryption strategy is developed by co‐multiplexing multicolor and hydrochromic luminescence of Sb‐doped double perovskite powders (SDPPs). The Cs_2_NaInCl_6_:Sb, Cs_2_KInCl_6_:Sb, and Cs_2_AgInCl_6_:Sb powders synthesized through coprecipitation methods exhibited broadband emissions with bright blue, cyan, and orange colors, respectively. Then, a white light‐emitting diode (W‐LED) was fabricated by mixing the three SDPPs, which showed white color with superior quality. By combining different fluorescence signals of the three SDPPs, a multichannel information encryption strategy was developed. As a demonstration, an encrypted quick response (QR) code was composed by the three SDPPs. The correct information can only be decrypted with simultaneous presence of “UV lamp” and “632 nm filter”. Moreover, the three SDPPs exhibited different water‐sensitive hydrochromic properties. Different from the reversible change from blue to yellow luminescence in Cs_2_NaInCl_6_:Sb, Cs_2_KInCl_6_:Sb showed an irreversible change from cyan to yellow after humidification. Whereas, luminescence color of Cs_2_AgInCl_6_:Sb was relatively stable under moisture treatment. Based on the water‐triggered switchable luminescence behaviors, three different messages could be read out under different moisture conditions, which was used to further improve throughput and security of information.

## 2 Results and Discussion

**Figure 1:**
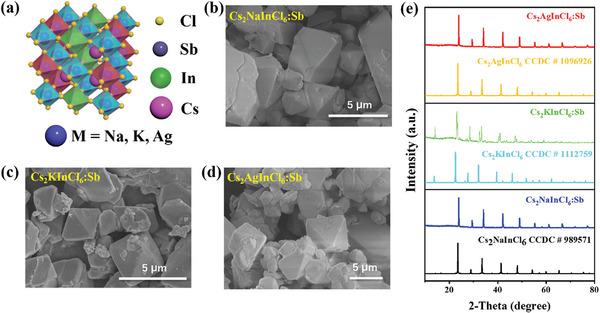
Structural characteristics of SDPPs. a) Schematic crystal structure of Cs_2_MInCl_6_:Sb double perovskites. b–d) SEM images of the b) Cs_2_NaInCl_6_:Sb, c) Cs_2_KInCl_6_:Sb, and d) Cs_2_AgInCl_6_:Sb. e) XRD patterns of the three SDPPs.

LHDP, Cs_2_MInCl_6_, shows a great potential as a luminescent material. Nonetheless, due to the parity‐forbidden transition restriction, Cs_2_MInCl_6_ powders usually exhibit weak PL. Hence, different approaches have been explored to improve the PL efficiency of Cs_2_MInCl_6_ powders. Among them, doping Sb^3+^ ions is one of the most effective ways to control over the electronic and optical properties of LHDPs.^[^ ^32^, ^33^, ^34^, ^35^ ^]^ As shown in **Figure** **1**a, Cs_2_MInCl_6_:Sb crystallizes in cubic system with Fm $\bar{3}$ m symmetry. The dopants of Sb^3+^ are prone to partially occupy the octahedral In^3+^ site due to their similar ionic radius and valence state. Here, the Cs_2_MInCl_6_:Sb (M = Na, K, Ag) powders were synthesized via a coprecipitation method.^[^ ^36^, ^37^, ^38^ ^]^ Scanning electron microscopic (SEM) images of the SDPPs are shown in Figure 1b–d. Most of the particles have a typical octahedral shape with sizes of ≈1–5 microns. The Sb^3+^ with smaller ion radius (76 pm) replaces the In^3+^ with larger ion radius (80 pm), resulting in the tiny lattice shrinkage. As shown in Figure 1e, after Sb^3+^ ion doping at a low concentration, the diffraction peak position slightly shifts to higher degree compared to that of pristine Cs_2_MInCl_6_ (M = Na, K, Ag), suggesting the successful doping.

**Figure 2:**
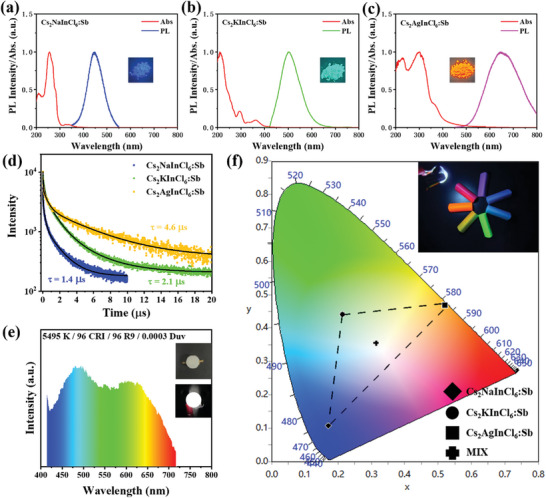
Luminescence characteristics of SDPPs and applications in white light illumination. a–c) Normalized absorption and PL spectra of a) Cs_2_NaInCl_6_:Sb, b) Cs_2_KInCl_6_:Sb, and c) Cs_2_AgInCl_6_:Sb. Inset: photographs of the SDPPs excited at 365 nm UV lamp. d) Time‐resolved PL decay curves of the SDPPs. e) Electroluminescence (EL) spectra of a W‐LED by using the SDPPs as color convertor. The inset shows the corresponding daylight and EL photograph of the W‐LED. e) CIE coordinates of three SDPPs and W‐LED. The inset shows the lighting capacity of the LED.

As extensively reported previously, in double perovskites, the strong exciton–lattice interaction leads a transient elastic Jahn–Teller lattice distortion due to the soft lattice, resulting in a change of nuclear coordinate and dissipation of exciton energy.^[^ ^39^, ^40^, ^41^, ^42^ ^]^ Thus, the PL of LHDPs is dominated by the recombination of self‐trapped excitons (STE) caused by the strong exciton–lattice coupling, which usually yields the broadband emission (ca.80–120 nm) with a large Stokes shift (ca.0.8–1.2 eV) and long PL lifetime (microsecond). The PL and absorption spectra of the three SDPPs are shown in **Figure** **2**a–c. The optical absorption is associated with the s^2^ to s^1^p^1^ transition of Sb^3+^ ion, including the transitions from ground state ^1^S_0_, to the singlet state ^1^P_1_, and the triplet state ^3^P_n_, where *n* = 1, 2, 3. The ^1^S_0_‐^1^P_1_ transition is allowed, causing the C band at ≈250–265 nm. The absorption band, located at ≈275–295 nm (B band), is assigned to the parity and spin forbidden ^1^S_0_ → ^3^P_2_ transition, which is relaxed by vibronic coupling and spin−orbit coupling. The ^1^S_0_‐^3^P_1_ transition (A band) is only partially allowed due to spin−orbit coupling for heavy atoms, which split due to a dynamic Jahn−Teller distortion of the excited state (320–360 nm). The absorption spectra of Cs_2_KInCl_6_:Sb and Cs_2_NaInCl_6_:Sb are very similar except for the slightly stronger A band of Cs_2_KInCl_6_:Sb than that of Cs_2_NaInCl_6_:Sb. Whereas, the absorption edge of Cs_2_AgInCl_6_:Sb remarkably redshifts compared to the other two, indicating the further enhanced A band caused by much stronger Jahn−Teller distortion.

All the three SDPPs exhibit relative broadband PL with large Stokes shift, in agreement with characteristic of STE emission. Whereas, the PL peak positions Cs_2_NaInCl_6_:Sb, Cs_2_KInCl_6_:Sb, and Cs_2_AgInCl_6_:Sb appear at 440, 510, and 640 nm, respectively (Figure 2a–c). The photon energy (*E_PL_*) of STE emission can be determined according to the following equation.

(1) $$E_{PL}=E_{g}\;-E_{b}-E_{ST}-E_{d}$$

where, Eg, Eb, E_ST_, and E_d_ represent the semiconductor bandgap, exciton binding energy, self‐trapping energy, and lattice distortion energy, respectively. As revealed by the XRD, the change of M^+^ sites can scarcely tune crystal lattice parameter. Thus, it is challenge to largely tune the semiconductor bandgap directly. However, the values of E_ST_ and E_d_ depend on the octahedral structure. When different elements are selected as M^+^, octahedral structure of [MCl_6_]^5−^ in the crystal changes. From Na^+^, K^+^, to Ag^+^, the ionic radius gradually increases, implying the easier and larger degree of Jahn−Teller distortion. As a result, the values of E_ST_ and E_d_ increase. Thus, the PL peak position redshifts. Besides, the full width at half maxima (FWHM) of the Cs_2_KInCl_6_:Sb, Cs_2_NaInCl_6_:Sb, and Cs_2_AgInCl_6_:Sb are 80, 100, and 120 nm, respectively, indicating the gradually increased exciton–lattice coupling, which is in agreement with the gradually enhanced A band observed in absorption spectra.

The PL excited at different wavelengths and PL excitation (PLE) spectra for each SDPPs are shown in Figures S1–S3 (Supporting Information). No significant shift is found in the PL spectra at various excitation wavelengths, which excludes that the existence of multiple emissive centers in a single SDDP (Figures S1–S3, Supporting Information). All the PLE spectra mainly contains three bands that correspond to B band (275–295 nm) and split A band (320–360 nm) observed in the absorption spectra. The PLE spectrum of Cs_2_AgInCl_6_:Sb contains a strong band at 380 nm, which is in agreement with the absorption spectrum. Figure 2d shows the time‐resolved PL (TRPL) decay curves of three SDPPs, which are fitted by tri‐exponential decay functions. The fitting details are presented in Table S1 (Supporting Information). The average decay lifetimes of Cs_2_NaInCl_6_:Sb, Cs_2_KInCl_6_:Sb, and Cs_2_AgInCl_6_:Sb are determined to be 1.4, 2.1, and 4.6 µs. The gradually increased PL lifetime is in consistence with the increased degree of Jahn−Teller distortion. Such long PL lifetimes at microsecond scale conform to the STE recombination mechanism.

With the three SDPPs, we obtained three broadband luminescence locating at different wavelengths, which almost cover the entire visible range. Thus, high‐quality white luminescence can be achieved by combining the three SDPPs. To verify this kind of feasibility, the three SDPPs were uniformly mixed and packaged by polydimethylsiloxane (PDMS), which was then installed on a 365 nm‐InGaN LED chip to fabricate a W‐LED. To avoid interference from the leaked UV light, a 420 nm long‐pass filter is added during the spectral measurement. Meanwhile, a 720 nm short‐pass filter is added to remove the second‐order diffraction of the leaked UV light. Emission spectrum of the W‐LED is shown in Figure 2e, which almost includes all the colors. Thus, a high color‐rendering index of 96 is achieved. The Commission International de l'Eclairage (CIE) coordinates of three SDPPs and the W‐LED module are shown in Figure 2f. CIE coordinate of W‐LED locates at (0.3152, 0.3537), which is very close to the standard white light. The high‐quality white light emission shows great application prospects in lighting and display devices.

**Figure 3:**
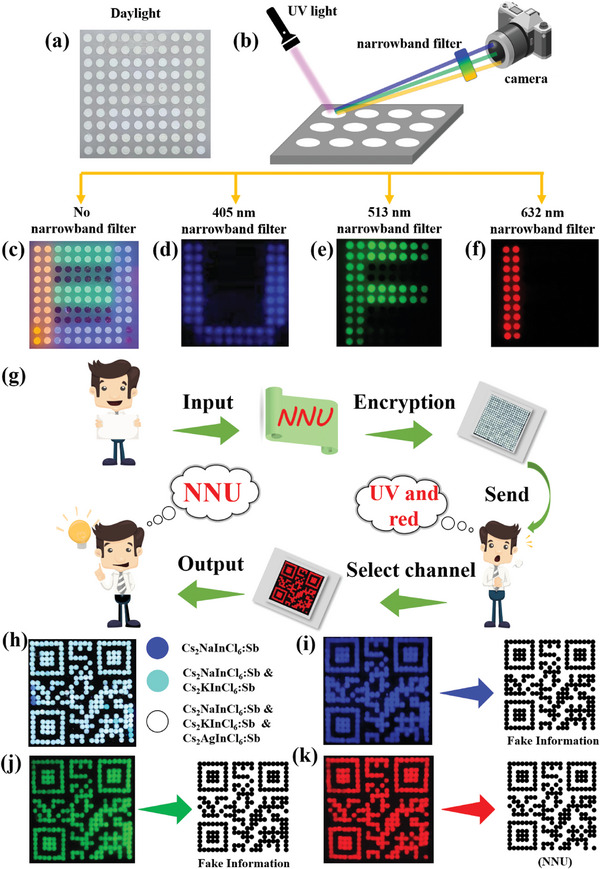
Optical information encryption based on multicolor luminescence from three types of SDDP@PDMs. a) Daylight photo of an array formed by using the SDDP@PDMs as pixels. b) Schematic illustration of the information encryption strategy. c–f) Luminescent pixels captured without filter c), with 405 nm filter d), with 513 nm filter e), with 632 nm filter f). g) Schematic illustration of the encryption and decryption on QR code. h–k) Fluorescent patterns observed under different channels and their corresponding QR code information. h) Distribution diagram of SDDP@PDMs, i) 405 nm filter (Fake information), j) 513 nm filter (Fake information), k) 632 nm filter (NNU).

Moreover, the multi‐color PL of the SDPPs under excitation at 365 nm also enables optical information encryption by color‐multiplexing. The information is delivered by different types of SDPPs. When the three SDPPs are mixed, the information is encrypted. The concealed information can be read out by adding narrowband filters. The information corresponding to Cs_2_NaInCl_6_:Sb can be identified by a 405 nm band filter, while the information delivered by Cs_2_KInCl_6_:Sb and Cs_2_AgInCl_6_:Sb can be resolved by 514 and 632 nm band filters, respectively. As shown in **Figure** **3**a, a 10 × 10 dot matrix is made by using the SDPPs/PDMS or BaSO_4_/PDMS as the pixels. Non‐fluorescent BaSO_4_ exhibiting white color that similar to SDPPs is used to represent blank pixel. The dot matrix does not transmit any information under sunlight. Figure 3b illustrates the information decryption method. The fluorescent photo under UV excitation displays unrecognizable and disordered information (Figure 3c). The letters “U”, “F”, and “I” can be easily read out by adding 405, 514, and 632 nm band filters, respectively (Figure 3d–f).

The broadband PL spectra of the three SDPPs imply the large spectral overlap between different SDPPs. Thus, it is hard to distinguish different SDPPs without the band filters, indicating a high security level. As a demo, this information encryption strategy is used to protect a QR code. As shown in Figure 3g, the QR code carrying the correct information (NNU) is delivered by Cs_2_AgInCl_6_:Sb, which is hidden in a 21 × 21 dot matrix. Under UV excitation, most of the pixels exhibit similar quasi‐white fluorescence due to the mixing of broadband PL although the components of them are different (Figure 3h). By adding 405, 514, and 632 nm band filters, three different fluorescent patterns can be obtained (Figure 3i–k). Thus, four different QR codes can be converted from the fluorescent patterns, i.e., white, blue, green, and red ones. Here, the correct information (“NNU”) only can be readout by scanning the red fluorescent pattern obtained by a 632 nm band filter. The abundant interference information can significantly improve the encryption security of real information. Moreover, the SDPPs wrapped in PDMs have good photostability and long‐term stability (Figures S7 and S8, Supporting Information), the encrypted information can be maintained in ambient environment for more than 180 days (Figures S9 and S10, Supporting Information).

**Figure 4:**
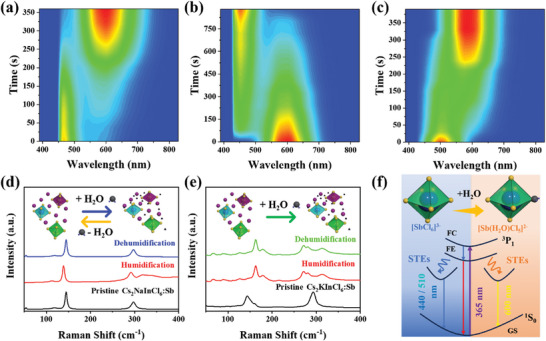
Water triggered discoloration behaviors of SDPPs. a) Humidification induced PL variation of Cs_2_NaInCl_6_:Sb. b) Dehumidification induced PL variation of Cs_2_NaInCl_6_:Sb. c) Humidification induced PL variation of Cs_2_KInCl_6_:Sb. d) Raman spectra of pristine, humidified, and dehumidified Cs_2_NaInCl_6_:Sb. e) Raman spectra of pristine, humidified, and dehumidified Cs_2_KInCl_6_:Sb. The illustration shows a schematic diagram of changes in crystal structure. f). Electron transition conformational coordinates of different PL in Cs_2_MInCl_6_:Sb (M = Na, K) before and after humidification.

In addition, the three SDPPs show special water triggered luminescence discoloration behavior. **Figure** **4**a shows the PL variation of Cs_2_NaInCl_6_:Sb after water spray. The PL peak at 450 nm gradually disappears while a broad band at 600 nm grows. This hydrochromic behavior is reversible after drying (Figure 4b). A similar PL variation is also observed for Cs_2_KInCl_6_:Sb (Figure 4c; Figure S11, Supporting Information). However, the hydrochromic behavior of Cs_2_KInCl_6_:Sb is irreversible. PL of Cs_2_AgInCl_6_:Sb keeps unchanged after water treatment (Figure S12, Supporting Information). To gain insight into the PL variation behavior, SEM, XRD, and XPS of the water‐treated Cs_2_KInCl_6_:Sb were measured (Figures S13 and S14, Supporting Information). SEM images shows the microcrystals keep octahedral shapes. XRD reveals the formation of (Cs/K)_2_InCl_5_(H_2_O):Sb (Figure S13, Supporting Information), which is supported by Raman spectral changes. As shown in Figure 4d, the Raman spectrum of pristine Cs_2_NaInCl_6_:Sb shows a 145 cm^−1^ and a band at 297 cm^−1^, which are ascribed to the longitudinal optical (LO) phonon modes from the symmetric stretching vibration of Cl atoms around Sb/In atoms in the octahedron. After humidification, the 297 cm^−1^ band splits due to the structural transformation from tetragonal Cs_2_NaInCl_6_:Sb to orthorhombic (Cs/Na)_2_InCl_5_(H_2_O) with lower symmetry.^[^ ^43^, ^44^ ^]^ The Raman spectrum is reversible after dehumidification, which agrees with the PL variation. Similar band splitting is also observed in the Raman spectrum of humidified Cs_2_KInCl_6_:Sb (Figure 4e). Whereas the Raman band splitting of (Cs/K)_2_InCl_5_(H_2_O):Sb is irreversible, in consistence with the irreversible PL variation.^[^ ^45^ ^]^ Thermogravimetric analysis also confirms the formation of hydrates (Figure S16, Supporting Information). It is worth mentioning that this type of hydrated SDDPs also has high stability (Figure S17, Supporting Information).

Following the above analyses, the excited‐state dynamics of Sb^3+^ in Cs_2_MInCl_6_:Sb (M = Na, K) can be schematically illustrated in a configuration coordinate diagram shown in Figure 4f. For the pristine Cs_2_NaInCl_6_:Sb, [InCl_6_]^3–^ in the structure is composed of six identical Cl^–^ ions and In^3+^ connections, so [InCl_6_]^3–^ exists in the form of a regular octahedron. When Sb^3+^ ions with ns^2^ electronic configuration are introduced, Sb^3+^ ions can perfectly replace In^3+^ to form [SbCl_6_]^3–^. Whereas, regarding the humified SDPPs, the octahedrons in the structure of (Cs/Na)_2_InCl_5_(H_2_O):Sb are [In(H_2_O)Cl_5_]^2–^ and [Sb(H_2_O)Cl_5_]^2–^, which are composed of five Cl^–^ ions, one H_2_O, and one In^3+^ (Sb^3+^) ion. This is equivalent to one H_2_O replacing one Cl^–^ in the regular octahedron, leading to the destruction of symmetry and subsequent Jahn–Teller distortion. The [Sb(H_2_O)Cl_5_]^2–^ octahedron in the (Cs/Na)_2_InCl_5_(H_2_O):Sb has a larger structural distortion than [SbCl_6_]^3–^ octahedron in the Cs_2_NaInCl_6_:Sb, resulting in a stronger Jahn–Teller distortion. The stronger Jahn–Teller distortion leads to a larger Stokes shift and a longer PL lifetime, thus the PL peak position redshifts and PL intensity increases (Figure 4a).^[^ ^46^ ^]^ The water‐triggered luminescence change of Cs_2_KInCl_6_:Sb can be explained similarly. Moreover, the concept of dimension in perovskite is usually referred the electronic dimension.^[^ ^37^, ^47^, ^48^ ^]^ In the crystal of water inserted double perovskite, the orbital overlap between the nearest octahedra is broken by H_2_O molecule, resulting in reduced electronic dimensionality. Thus, the (Cs/K)_2_InCl_5_(H_2_O) is usually designated as 0D structure.

**Figure 5:**
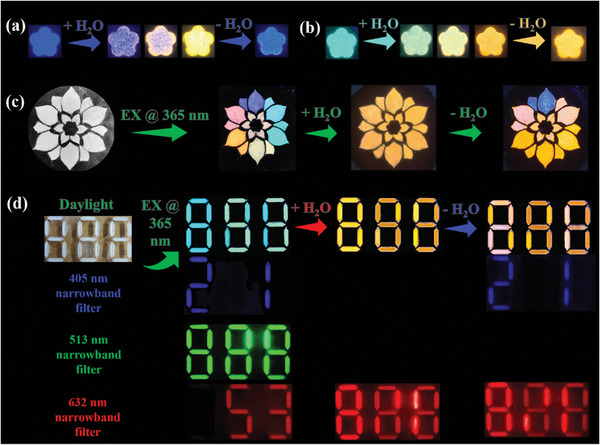
Information encryption based on water triggered discoloration of SDPPs. a,b) Fluorescent photos of a) Cs_2_NaInCl_6_:Sb and b) Cs2KInCl_6_:Sb during humidification and dehumidification. c) Color changes of multicolor anti‐counterfeiting flowers composed of Cs_2_NaInCl_6_:Sb and Cs_2_KInCl_6_:Sb after humidification and dehumidification. g) Digital encryption strategy that combines the water triggered color change characteristics of SDPPs with optical channel selection.

Based on the above discussions, the three SDPPs exhibit three distinctive water triggered luminescence variation behaviors. **Figure** **5**a shows reversible PL transition of Cs_2_NaInCl_6_:Sb after moisture treatment and dehumidification, whereas, the hydrochromic process of Cs_2_KInCl_6_:Sb is irreversible (Figure 5b). As shown in Figure 5c, a flower composed of Cs_2_NaInCl_6_:Sb, Cs_2_KInCl_6_:Sb, and (Cs/K)_2_InCl_5_(H_2_O):Sb exhibits polychromatic luminescence at the initial stage. For example, a mixture of blue Cs_2_NaInCl_6_:Sb and yellow (Cs/K)_2_InCl_5_(H_2_O):Sb at ratio of 1:1 leads the pink petals. Whereas, mixing the Cs_2_NaInCl_6_:Sb, Cs_2_KInCl_6_:Sb, and (Cs/K)_2_InCl_5_(H_2_O):Sb in a 1:1:1 ratio can produce white petals. After water spray, color of all the petals becomes yellow due to the water triggered luminescence discoloration. After dehumidification, luminescence of the petals composed of Cs_2_NaInCl_6_:Sb recovers while other petals maintains yellow luminescence. Therefore, a single pattern can deliver three various luminescent information at different humified states, which can also be used for information encryption. As shown in Figure 5d, three digital patterns are formed by SDPPs. Under UV excitation, the three patterns display the indistinguishable number “888” due to mixed broadband PL. However, by using corresponding narrowband filters, the blue number “21”, green number “888”, and red number “53” can be easily recognized. After humidification, all the components emit yellow photons due to the hydrochromic behavior. Thus, no fluorescence can be observed with blue and green filters. With a red filter, a number “888” is observed. Therefore, the confidential information “21” “53” are totally concealed at the humified state. If “21” is defined as the real information, the confidential information can be switched between concealed and displayed states through a simple water spray/drying. Whereas, when “53” collected from the green channel is the real information, the confidential information can be permanently erased, fundamentally avoiding information leakage. Therefore, different encryption schemes can be flexibly designed based on SDPPs to meet a variety of encryption requirements. Last but not the least, PL quantum yields (PLQYs) of SDPPs were measured (Figures S19 and S20, Supporting Information). The PLQYs of Cs_2_MInCl_6_ (M = Na, K, Ag) are ≈5%. After humidification, the PL intensity remarkably increases to ≈20%, which are in good agreement with the STE mechanism as discussed above. These PLQYs are slightly lower than the state‐of‐ art level. Nevertheless, the luminescence is strong enough to be observed by naked eyes, which is sufficient for fluorescence pattern. For practical applications, the composition and reaction conditions can be further optimized to increase the PLQY. For example, the PL intensity can be increased by adjusting the Sb^3+^ doping concentration (Figure S21, Supporting Information).

## 3 Conclusion

In summary, we successfully synthesized three types of SDPPs through a simple coprecipitation method. Profiting from their multicolor STE emissions and broad spectra, a W‐LED based on the mixture of SDPPs produced a high‐quality white emission with CRI as high as 96. In addition, a multi‐channel optical information encryption strategy was developed by mixing the SDPPs. The different mixture of SDPPs usually exhibit similar quasi‐white fluorescence, thus the information delivered by a specific type of SDPPs is concealed. Narrowband filters can serve as the keys to unveil the corresponding encrypted information. An encrypted QR code is demonstrated based on this strategy. We would like to emphasize that there is normally a trade‐off between information security and verification convenience. Among the existing techniques to increase security, the use of filters is relatively convenient and cheap. Moreover, we reveal the three types of SDPPs exhibit three distinguishable water‐triggered luminescence variations. The luminescence of Cs_2_NaInCl_6_:Sb shows reversible hydrochromic behaviors under humidification and dehumidification. Whereas, the luminescence variation of Cs_2_KInCl_6_:Sb is irreversible and the luminescence of Cs_2_AgInCl_6_:Sb is relatively stable. Therefore, a single pattern based on the SDPPs can deliver various luminescent information at different humidified states. The simultaneous multi‐channel and water triggered switchable luminescence lay the foundation for the future design of advanced information encryption.

## 4 Experimental Section

### Materials

HCl (Sinopharm Chemical Reagent, 37%), SbCl_3_ (Macklin, 99.9%), CsCl (Aladdin, 99.9%), NaCl (Aladdin, 99%), K_2_O_3_ (Aladdin, 99.9%), C_2_H_3_O_2_Ag (Macklin, 99.5%), InCl_3_ (Aladdin, 99.99%), ethanol anhydrous (Sinopharm Chemical Reagent, 99.5%), and polydimethylsiloxane (PDMS). All chemicals were directly used without further purification.

### Synthesis of Sb‐Doped Double Perovskite Powders

For the synthesis of Cs_2_NaInCl_6_:Sb, SbCl_3_ (0.0114 g, 0.05 mmol), NaCl (0.0292 g, 0.5 mmol), InCl_3_ (0.0995 g, 0.45 mmol), and HCl (4 mL) were added to a round‐bottom flask and heated to 80 °C in an oil bath. Upon full solvation of these precursors, CsCl (0.1683 g, 1 mmol) was added, leading to the immediate formation of a white precipitate. The solution was left to stir for an additional 20 min before removing it from the oil bath and allowing the product to cool to room temperature to ensure the complete reaction of precursors. The precipitate was then filtered using a porous fritted funnel, washed several times with anhydrous ethanol, and dried overnight via vacuum filtration. The resulting powder was ground for 30 min and heated in an oven at 210 °C for 10 h to ensure homogenous Sb incorporation.

The synthesis of Cs_2_MInCl_6_:Sb (M = K, Ag) were conducted in similar procedures, while SbCl_3_ (0.0114 g, 0.05 mmol), K_2_O_3_ (0.0691 g, 0.5 mmol), C_2_H_3_O_2_Ag (0.0834 g, 0.5 mmol), and InCl_3_ (0.0995 g, 0.45 mmol) were used to form precursors. Then CsCl (0.1683 g, 1 mmol) was added when the precursors reached 80 °C.

### Preparation of SDPPs@PDMS Composites

First, 0.05 g SDPPs were mixed with 2 g PDMS, which were stood for 1 h until the bubbles completely disappear. Then, the product was poured into the mold and dried in an oven at 50 °C for 3 h.

### Characterizations

The diffuse reflectance spectroscopy data were obtained by using Shimazu UV2600 UV–vis spectrophotometer. And the diffuse reflectance spectra were converted into pseudo‐absorption by Kubelka‐Munk (KM) function.^[^ ^49^ ^]^ The steady‐state photoluminescence (PL) spectra were obtained by a Maya 2000 Pro high‐sensitivity spectrometer (Ocean optics) equipped on an optical microscope. A FM‐4P‐TCSPC fluorescence spectrometer was used to measure the photoluminescence excitation (PLE) and decay curves. D/max 2500/ PC rotating target X‐ray diffractometer was used to measure the X‐ray powder diffraction (XRD) patterns. Scanning electron microscope (SEM) measurements were performed on Thermo Scientific TM Apreo 2s, acceleration voltage working at 5 kV. X‐ray Photoelectron Spectroscopy (XPS) were obtained by a ESCALAB Xi+ electron spectrometer.

## Conflict of Interest

The authors declare no conflict of interest.

## Supporting information

Supporting Information

## Data Availability

The data that support the findings of this study are available from the corresponding author upon reasonable request.
