# Supplementary material for: Advanced Optical Information Encryption Enabled by Polychromatic and Stimuli‐Responsive Luminescence of Sb‐Doped Double Perovskites
Source: Adv Sci (Weinh). 2024 Apr 16;11(24):2308390. doi: 10.1002/advs.202308390 (PMC11200084; doi:10.1002/advs.202308390)
Supplement: Supplementary file 1 — Supporting Information [file ADVS-11-2308390-s001.pdf]

ADVANCED SCIENCE
Open Access

Supporting Information

for *Adv. Sci.*, DOI 10.1002/advs.202308390

Advanced Optical Information Encryption Enabled by Polychromatic and Stimuli-Responsive Luminescence of Sb-Doped Double Perovskites

Yijun Zhao, Xingru Yang, Lun Yang, Fangjian Xing, Cihui Liu, Yunsong Di, Guiyuan Cao, Shibiao Wei, Xifeng Yang, Xiaowei Zhang, Yushen Liu and Zhixing Gan**

Supporting Information

Advanced Optical Information Encryption Enabled by Polychromatic and Stimuli-Responsive Luminescence of Sb-doped Double Perovskites

*Yijun Zhao,[1] Xingru Yang,[1] Lun Yang,[2] Fangjian Xing,[1] Cihui Liu,[1] Yunsong Di,[1] Guiyuan Cao,[3] Shibiao Wei,[3] Xifeng Yang,[4] Xiaowei Zhang,[5] Yushen Liu,[4] and Zhixing Gan[1]**

1. Center for Future Optoelectronic Functional Materials, School of Computer and Electronic Information/School of Artificial Intelligence, Nanjing Normal University, Nanjing 210023, China

2. Institute for Advanced Materials, Hubei Key Laboratory of Pollutant Analysis & Reuse Technology, Hubei Normal University, Huangshi 435002, China

3. Nanophotonics Research Center, Shenzhen Key Laboratory of Micro-Scale Optical Information Technology, Shenzhen University, Shenzhen 518060, China

4. College of Electronic and Information Engineering, Changshu Institute of Technology, Suzhou 215500, China

5. Department of Electrical Engineering and Computer Science, Ningbo University, Ningbo 315211, China

*Corresponding author email address: zxgan@njnu.edu.cn

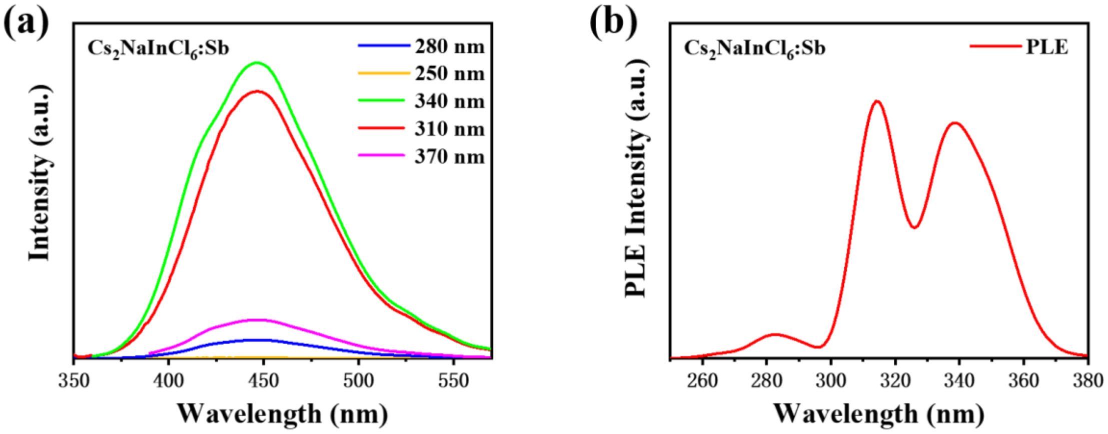

Figure S1. (a) PL spectra of $Cs_2NaInCl_6$:Sb excited at different wavelengths. (b) PLE spectra of $Cs_2NaInCl_6$:Sb.

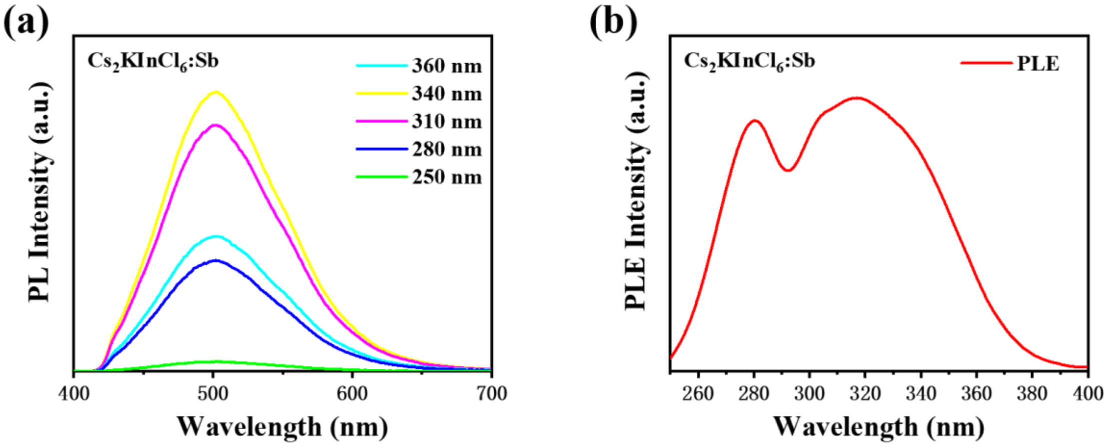

Figure S2. (a) PL spectra of Cs_2KInCl_6:Sb excited at different wavelengths. (b) PLE spectra of Cs_2KInCl_6:Sb.

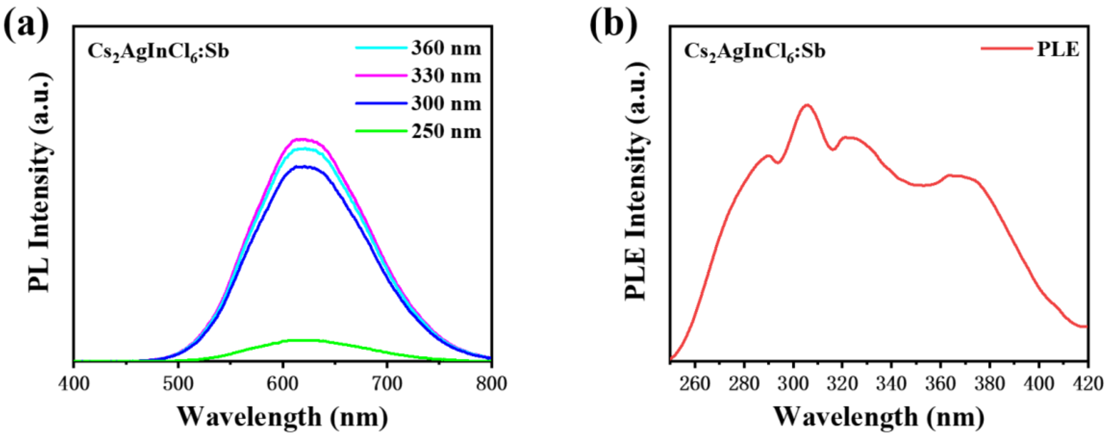

Figure S3. (a) PL spectra of $Cs_2AgInCl_6$:Sb excited at different wavelengths. (b) PLE spectra of $Cs_2AgInCl_6$:Sb.

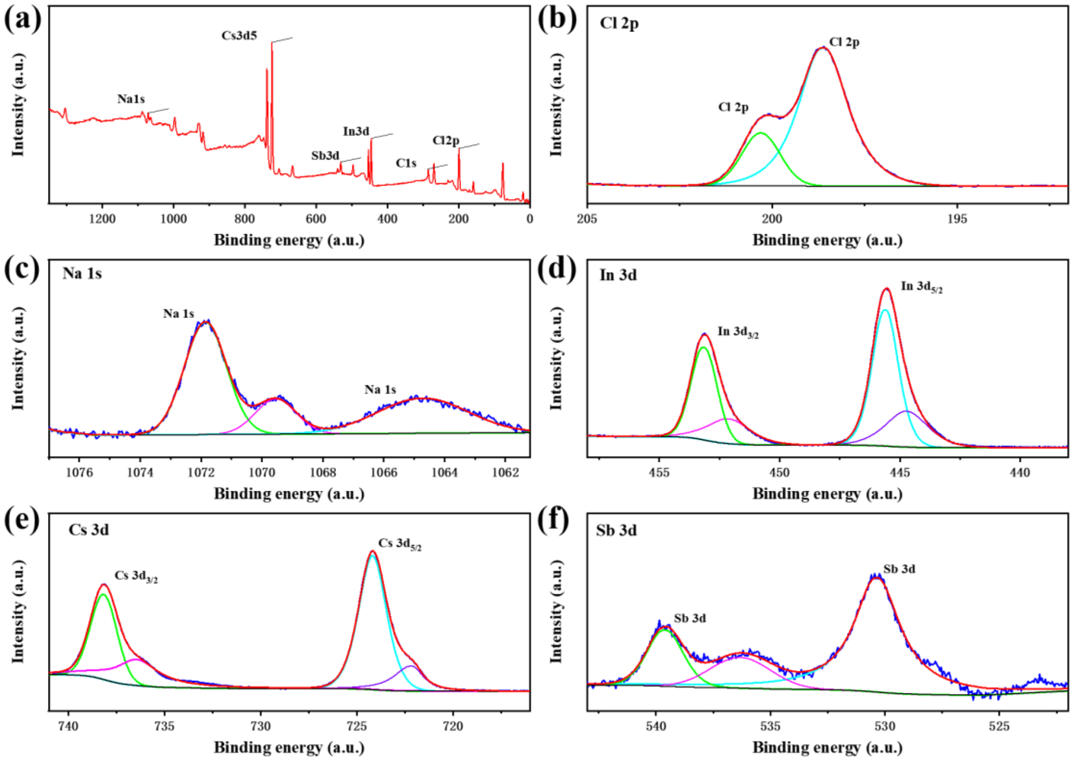

Figure S4. XPS spectra of the $Cs_2NaInCl_6$:Sb. (a) Full survey spectrum. (b-f) Cl 2p (b), Na 1s (c), In 3d (d), Cs 3d (e), Sb 3d (f).

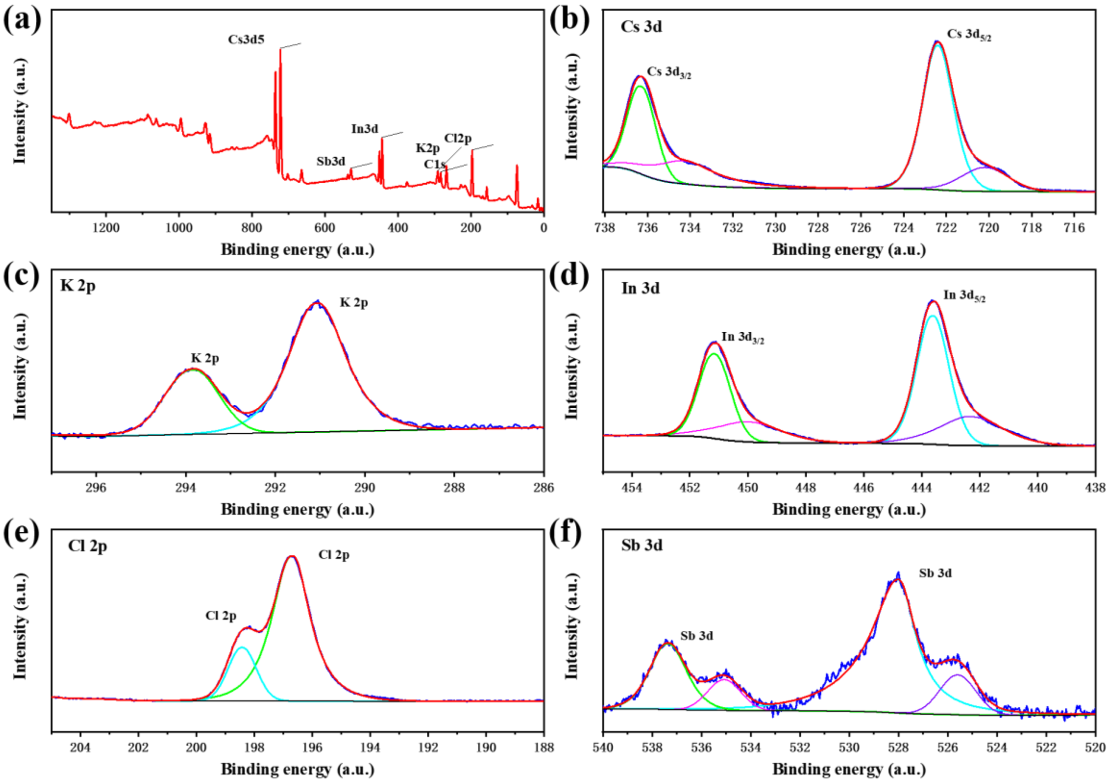

Figure S5. XPS spectra of the Cs_2KInCl_6:Sb. (a) Full survey spectrum. (b-f) Cl 2p (b), K 2p (c), In 3d (d), Cs 3d (e), Sb 3d (f).

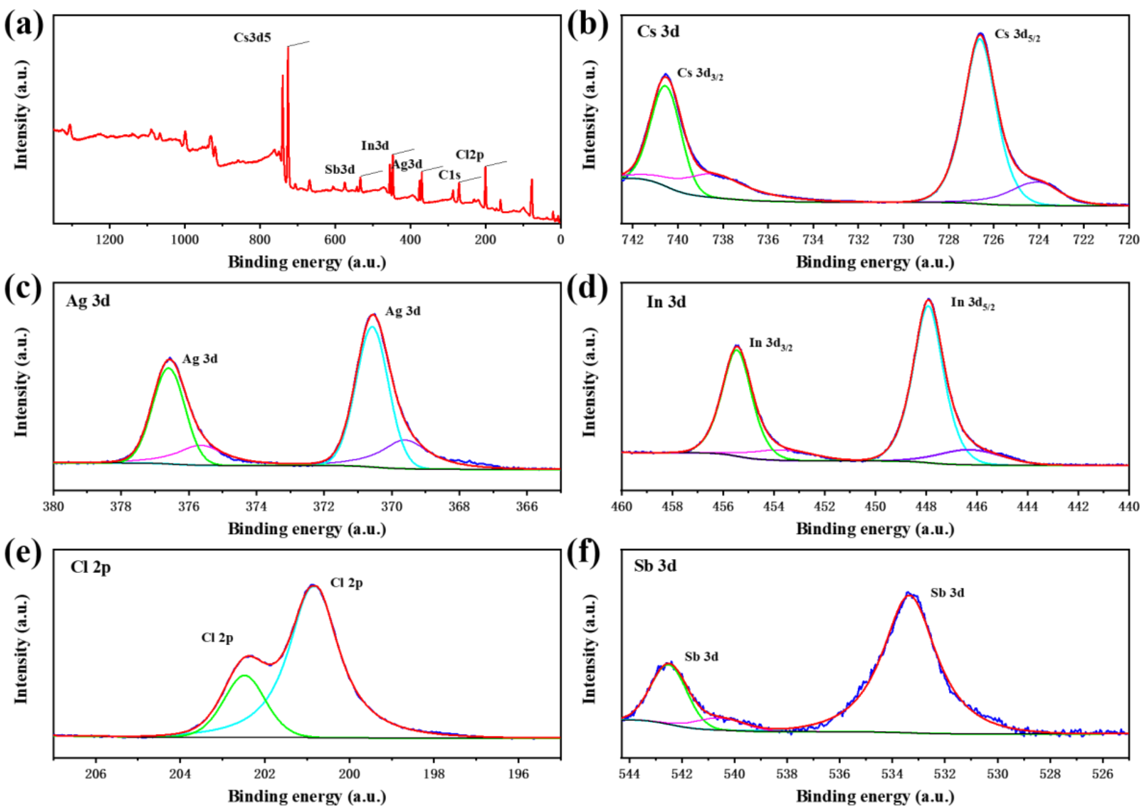

Figure S6. XPS spectra of the $Cs_2AgInCl_6$:Sb. (a) Full survey spectrum. (b-f) Cl 2p (b), Ag 3d (c), In 3d (d), Cs 3d (e), Sb 3d (f).

Table S1. Fitting details for the time-resolved PL decay curves.

Sample	A_1	τ_1	A_2	τ_2	A_3	τ_3
$Cs_2NaInCl_6$:Sb	2460.41627±24.05841	0.02344±4.4842E-4	1832.61572±13.77117	0.23887±0.00319	1096.15054±8.41843	1.7086±0.01395
Cs_2KInCl_6:Sb	3450.66915 ± 28.46197	0.07074 ± 0.00112	2970.89966 ± 22.19724	0.51062 ± 0.00741	2696.85171 ± 21.06532	2.4175 ± 0.01352
$Cs_2AgInCl_6$:Sb	6.11558E-7 ± 1.10414E-13	0.01904 ± 5.6468E-4	3901.03116 ± 2.56437	0.53383 ± 0.00962	2235.11846 ± 1.43815	5.34147 ± 0.06767
$(Cs/K)_2InCl_5(H_2O)$:Sb	3640.3528 ± 38.33748	0.02453 ± 4.8245E-4	2899.39309 ± 17.47351	0.31211 ± 0.02089	2937.27224 ± 7.02634	3.41221 ± 0.02089

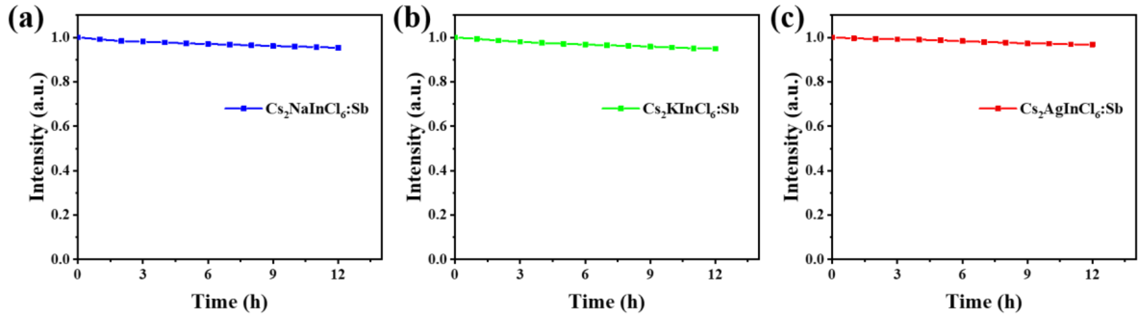

Figure S7. PL intensity of $Cs_2NaInCl_6$:Sb (a), Cs_2KInCl_6:Sb (b), $Cs_2AgInCl_6$:Sb (c) under continuous irradiation. The PL intensity was monitored under 365 nm UV lamp with a power of 5W.

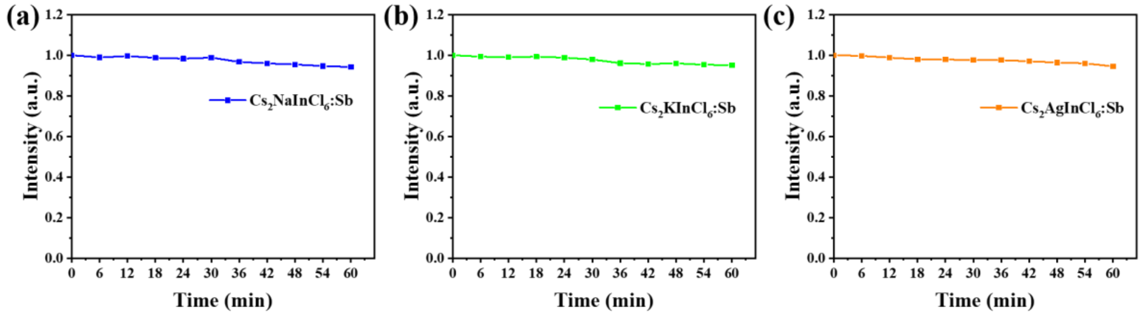

Figure S8. Stability of $Cs_2NaInCl_6$:Sb@PDMS (a), Cs_2KInCl_6:Sb@PDMS (b) and $Cs_2AgInCl_6$:Sb@PDMS (c) over spray treatment. The PL intensity was recorded at a time interval of 6 minutes.

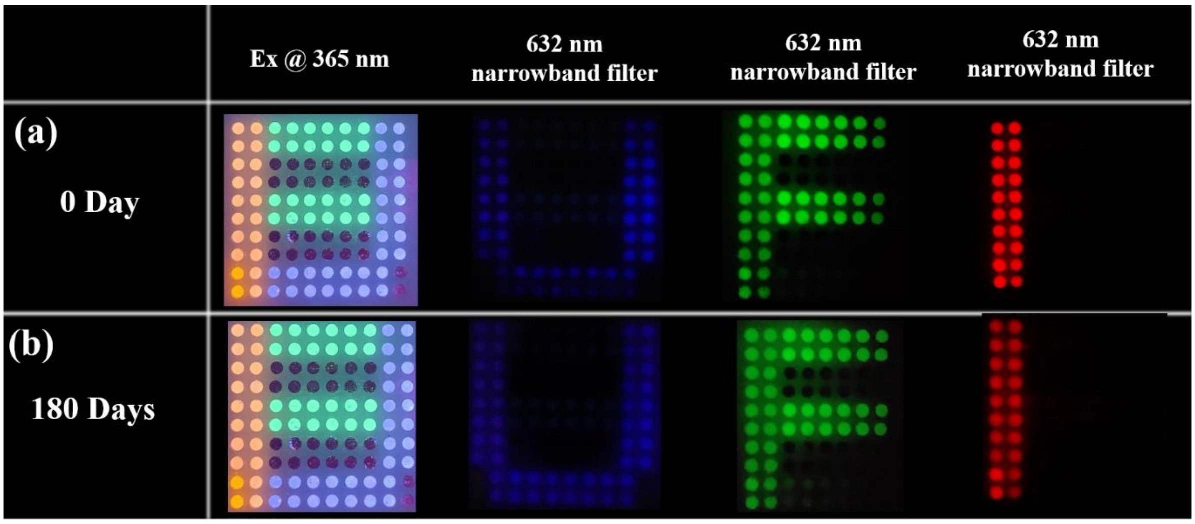

Figure S9. Pattern of a lattice formed by the SDPPs after storage for 0 (a), and 180 days (b), optical channels were selected at 410 nm, 365 nm and 254 nm narrowband filter.

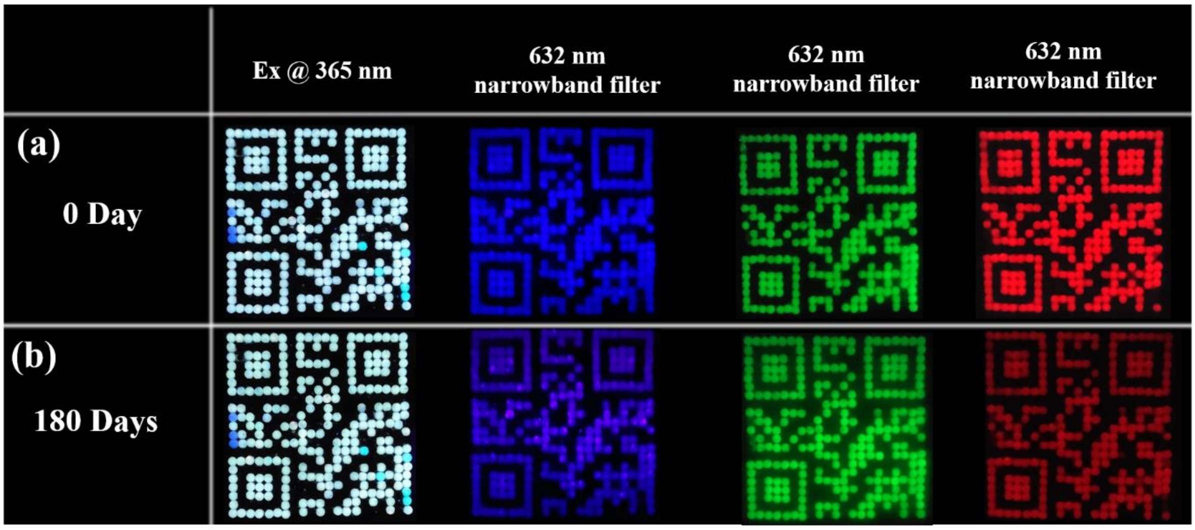

Figure S10. Pattern of a QR code formed by the SDPPs after storage for 0 (a), and 180 days (b). After storage in the ambient environment for 180 days, the PL intensities of the SDPPs almost keep constant, indicating the outstanding long-term stability with the encapsulation.

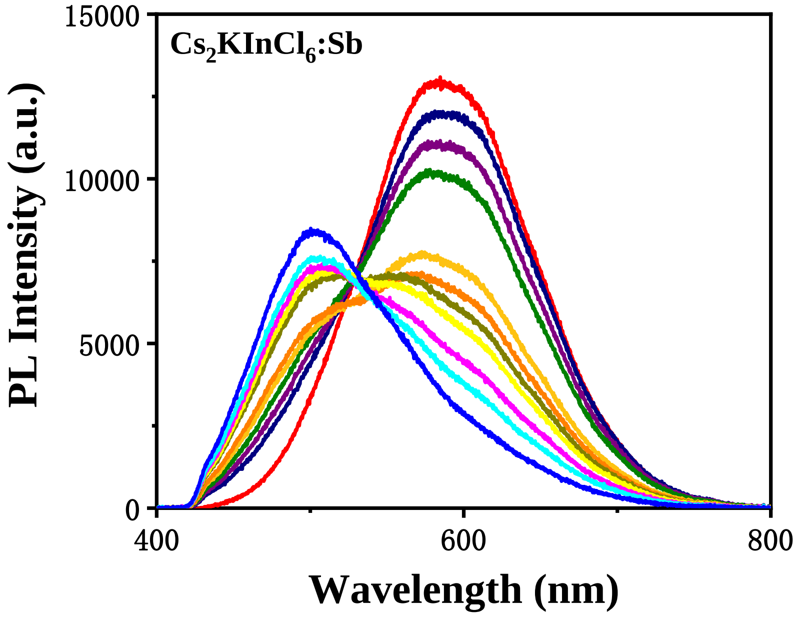

Figure S11. Humidification induced PL variation of Cs_2KInCl_6:Sb. The PL spectra were continuously recorded at a time interval of one minute after the spray treatment.

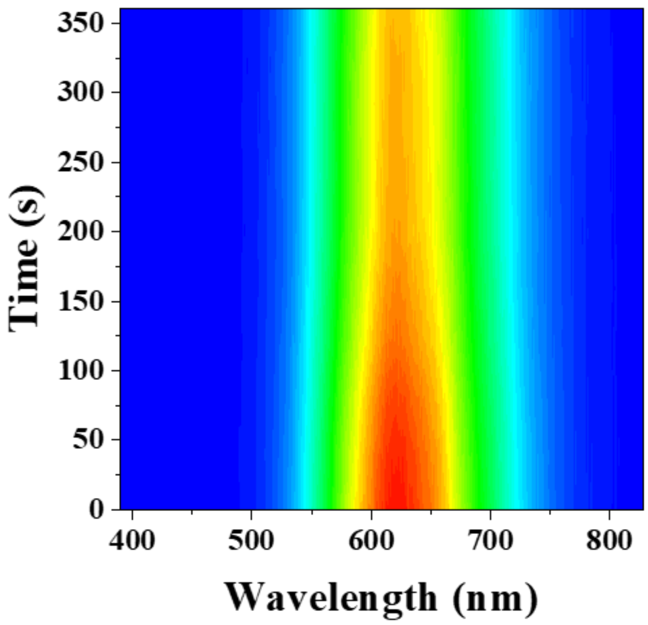

Figure S12. Humidification induced PL variation of $Cs_2AgInCl_6$:Sb.

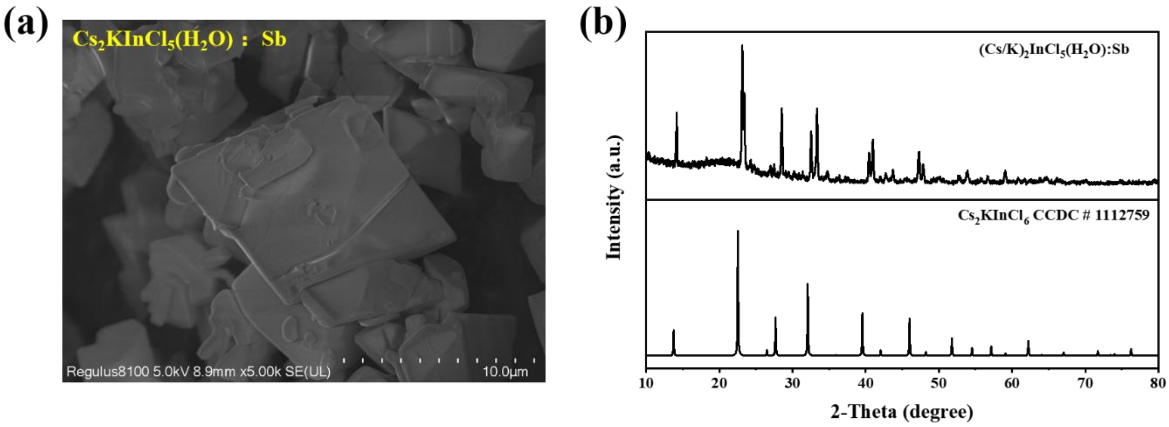

Figure S13. (a) SEM image of $(Cs/K)_2InCl_5(H_2O)$:Sb. (b) XRD patterns of $(Cs/K)_2InCl_5(H_2O)$:Sb.

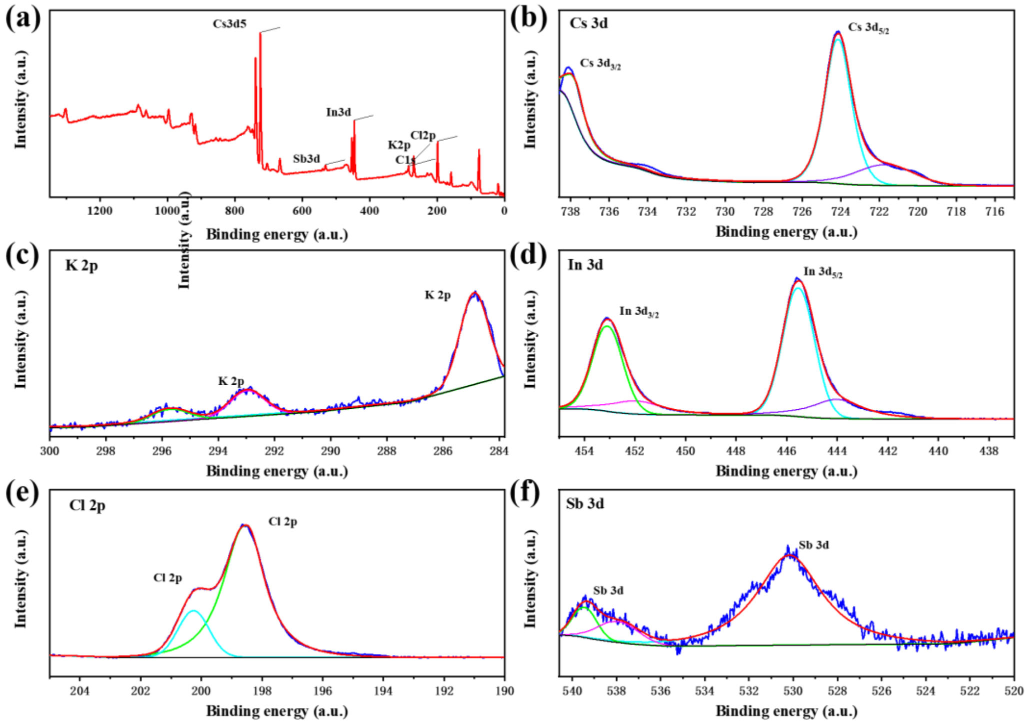

Figure S14. XPS spectra of $(Cs/K)_2InCl_5(H_2O)$. (a) Full survey spectrum. (b-f) Cl 2p (b), K 2p (c), In 3d (d), Cs 3d (e), Sb 3d (f).

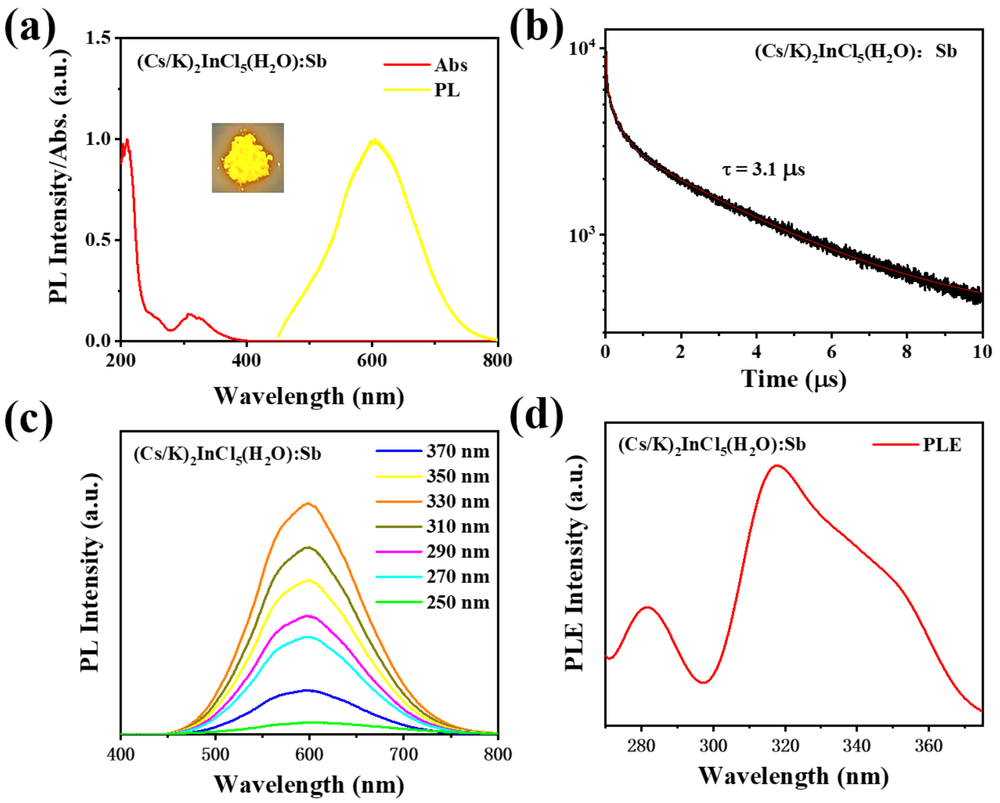

Figure S15. (a) Normalized absorption and PL spectra of $(Cs/K)_2InCl_5(H_2O)$. (b) Time-resolved PL decay of $Cs/K)_2InCl_5(H_2O)$. (c) PL spectra of $(Cs/K)_2InCl_5(H_2O)$ excited at different wavelengths. (d) PLE spectrum of $(Cs/K)_2InCl_5(H_2O)$:Sb.

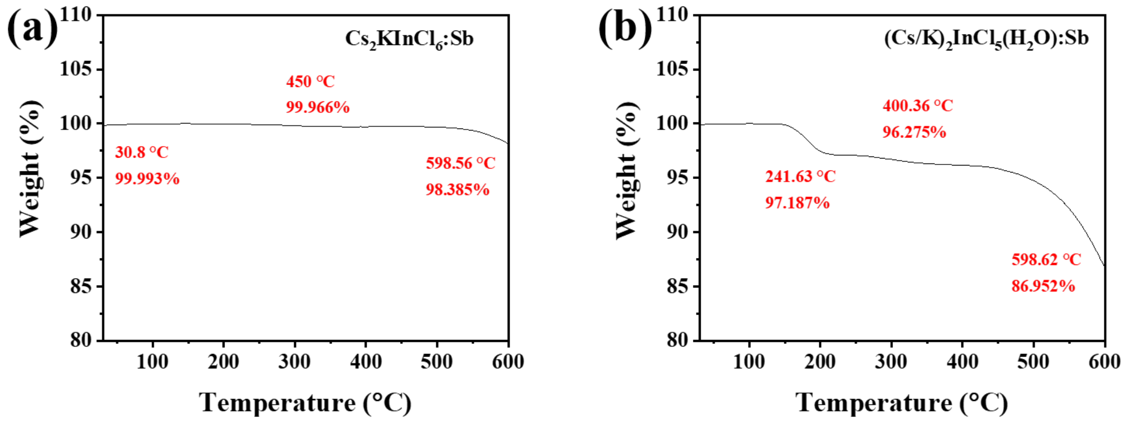

Figure S16. Thermogravimetric analysis (TGA) results of Cs_2KInCl_6:Sb (a) and $(Cs/K)_2InCl_5(H_2O)$:Sb (b) measured at a heating rate of 10 ^oC/min. The TGA of $(Cs/K)_2InCl_5(H_2O)$:Sb shows a remarkable mass loss at about 97 ^oC, indicating the dehydration.

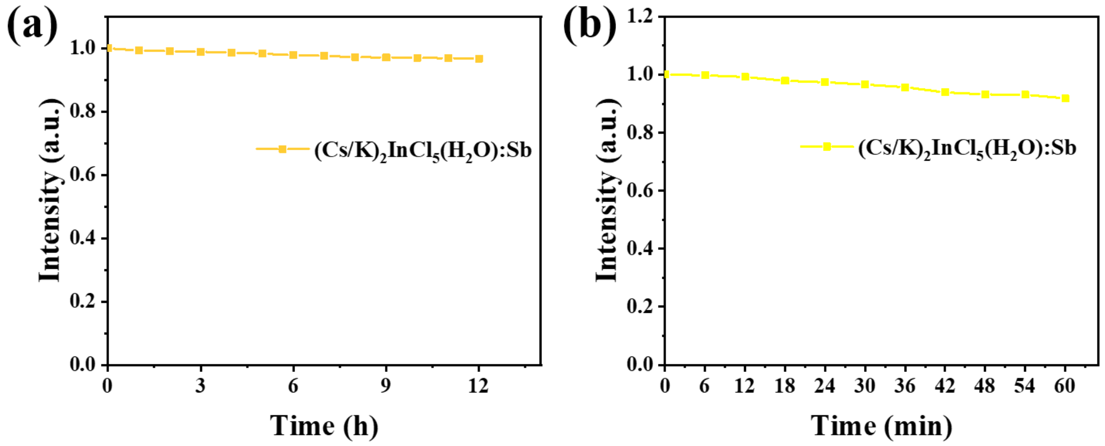

Figure S17. (a) PL intensity of $(Cs/K)_2InCl_5(H_2O)$:Sb under continuous UV irradiation. (b) PL intensity of $(Cs/K)_2InCl_5(H_2O)$:Sb @PDMS after spray treatment.

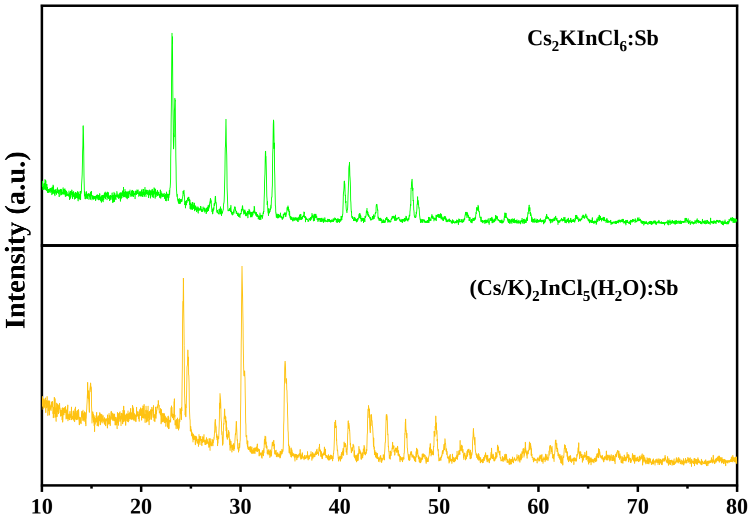

Figure S18. Comparison of XRD patterns of the Cs_2KInCl_6:Sb and $(Cs/K)_2InCl_5(H_2O)$:Sb. Compared to Cs_2KInCl_6:Sb, the XRD peak of the $(Cs/K)_2InCl_5(H_2O)$:Sb shifted and showed more bimodal peaks, due to the decrease in symmetry.

Table S2. Photoluminescence quantum yields of SDDPs.

Sample	PL quantum yield
$Cs_2NaInCl_6$:Sb	5.75 %
$(Cs/Na)_2InCl_5(H_2O)$:Sb	20.34 %
Cs_2KInCl_6:Sb	6.22 %
$(Cs/K)_2InCl_5(H_2O)$:Sb	21.94 %
$Cs_2AgInCl_6$:Sb	4.62%

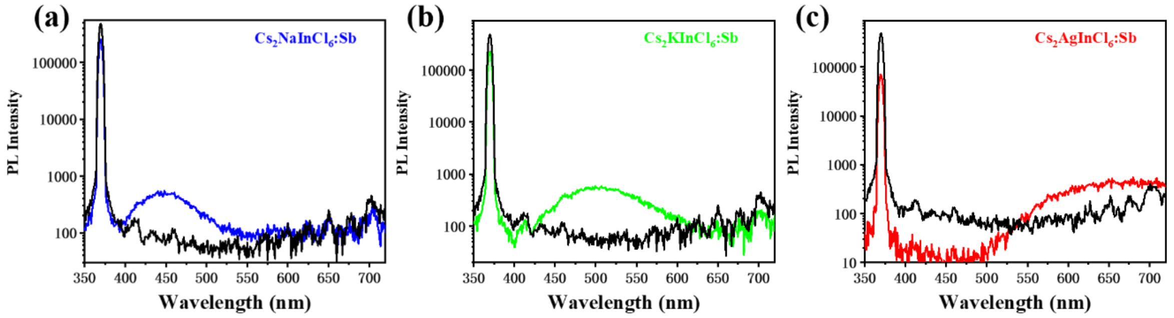

Figure S19. PL spectra of $Cs_2NaInCl_6$:Sb (a), Cs_2KInCl_6:Sb (b), $Cs_2AgInCl_6$:Sb (c) measured by integrating sphere for calculating PLQY. The black line is the reference spectrum of excitation beam.

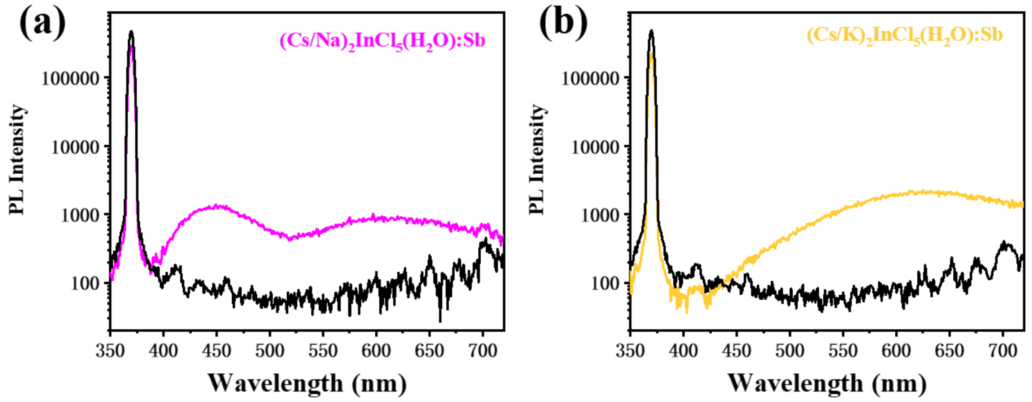

Figure S20. PL spectra of $(Cs/Na)_2InCl_5(H_2O)$:Sb. (a) and $(Cs/K)_2InCl_5(H_2O)$:Sb. (b) measured by integrating sphere for calculating PLQY. The black line is the reference spectrum of excitation beam.

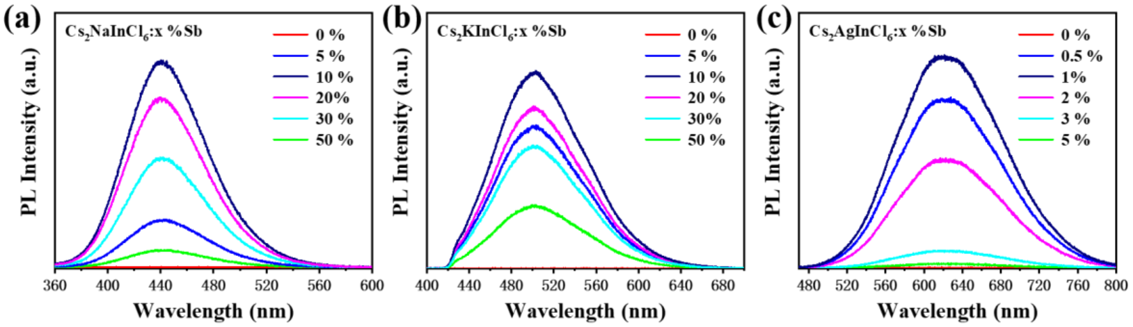

Figure S21. PL spectra of $Cs_2NaInCl_6$:x %Sb (a), Cs_2KInCl_6:x %Sb (b) and $Cs_2AgInCl_6$:x %Sb (c). The excitation wavelength is 320 nm.

The PL peak positions almost show no change with the incorporated Sb content. Whereas, the PL intensity increases first and then decreases with the increasing Sb/(Sb+In) feeding ratio. As well accepted, before Sb doping, STEs in Cs_2MInCl_6:Sb (M=Na, K, Ag) are dark states [Chemistry of Materials 2022, 34, 6288, Chemistry of Materials 2020, 32, 5118-5124, Advanced Functional Materials 2023, 33, 2212135]. After trace doping of Sb^{3+}, the emission intensity increased greatly while keeping the same peak position at 440/510/640 nm due to the transformation from dark to bright STEs [The Journal of Physical Chemistry Letters 2020, 11, 2053-2061, Chemistry of Materials 2020, 32, 5118-5124, Advanced Functional Materials 2023, 33, 2212135]. Higher Sb^{3+} doping concentration leads to a decrease in PL efficiency, which may be caused by a nonradiative recombination center from doping-induced defects[Adv. Funct. Mater.2023, 33, 2212135, Nano Res.2018, 11, 2104].
